# Diagnostic utility of the serum–ascites albumin gradient in Mexican patients with ascites related to portal hypertension

**DOI:** 10.1002/jgh3.12404

**Published:** 2020-08-08

**Authors:** Enrique Cervantes Pérez, Gabino Cervantes Guevara, Gabino Cervantes Pérez, Guillermo Alonso Cervantes Cardona, Clotilde Fuentes Orozco, Kevin Josué Pintor Belmontes, Bertha Georgina Guzmán Ramírez, Laura Lizeth Reyes Aguirre, Francisco José Barbosa Camacho, Aldo Bernal Hernández, Alejandro González Ojeda

**Affiliations:** ^1^ Department of Clinical Nutrition, Instituto Nacional de Ciencias Médicas y Nutrición Salvador Zubirán Universidad Nacional Autónoma de México Mexico City Mexico; ^2^ Department of Welfare and Sustainable Development University Center of the North, University of Guadalajara Colotlan Mexico; ^3^ Gastroenterology Departament Hospital Civil de Guadalajara “Fray Antonio Alcalde” University of Guadalajara Guadalajara Mexico; ^4^ Department of Philosophical, Methodological and Instrumental Disciplines Health Sciences University Center, University of Guadalajara Guadalajara Mexico; ^5^ Biomedical Research Unit 02 Western National Medical Center, Mexican Institute of Social Security Guadalajara Mexico

**Keywords:** albumin gradient, ascites, cirrhosis, portal hypertension, serum albumin–ascites gradient

## Abstract

**Background and Aim:**

Analysis of ascitic fluid is necessary to determine the etiology and to distinguish portal hypertension (PH)‐related and unrelated ascites. Numerous diagnostic parameters have been studied, but no single parameter has completely distinguished these. We aimed to validate the serum albumin–ascites gradient (SAAG) for the diagnosis of ascites secondary to PH and to establish cutoff points to predict PH using its sensitivity and specificity.

**Methods:**

This was a cross‐sectional study conducted on patients diagnosed with ascites of any etiology. The SAAG and albumin concentration in ascitic fluid (AFA) were measured to establish their sensitivity and specificity for determining the presence or absence of PH. Cutoff points and levels of statistical significance were established based on the area under the curve.

**Results:**

Eighty‐seven patients were evaluated, of whom 74 (84%) were men, with an average age of 54.0 ± 13.6 years. Seventy‐two (83%) were diagnosed at admission with PH‐related ascites and 15 (17%) with non‐PH‐related ascites. SAAG correctly classified 48 (67%) patients, but 24 (33%) were classified incorrectly, while AFA classified 59 (82%) correctly and only 13 (17%) incorrectly. The diagnostic accuracy of SAAG was 57 *versus* 73% for AFA. AFA had a sensitivity of 82% and specificity of 66% (95% confidence interval [CI]: 0.63–0.93), while SAAG had a sensitivity of 66% but a specificity of 86% (95% CI: 0.72–0.95).

**Conclusions:**

The SAAG showed poor diagnostic performance with low sensitivity but high specificity. The diagnostic accuracy of AFA is superior to that of SAAG in discriminating between PH and non‐PH ascites.

## Graphical abstract

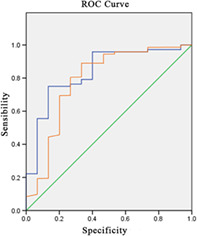

The serum albumin–ascites gradient (SAAG) and albumin concentration in ascitic fluid (AFA) were measured to establish their sensitivity and specificity for determining the presence or absence of portal hypertension (PH). The SAAG showed poor diagnostic performance with low sensitivity but high specificity. The diagnostic accuracy of AFA is superior to that of SAAG in discriminating between PH and non‐PH ascites.

## Introduction

Ascites is the pathological accumulation of fluid in the peritoneal cavity^1^, ^2^. The most common causes of ascites are parenchymal liver disease, followed by peritoneal malignancies, tuberculous peritonitis, congestive heart failure, nephrotic syndrome, and others (hypoalbuminemia, chylous ascites, Budd–Chiari syndrome, mixed ascites, and malnutrition).^1^, ^3^ The management of ascites depends on its etiology. Although several numerical diagnostic parameters have been investigated as biomarkers, no single parameter has been able to distinguish completely between etiologies, and hence, the search for a better marker continues.^3^, ^4^ Traditionally, the etiology of ascites was classified based on the transudate and exudate concept based on a cutoff of ˃2.5 mg/dL total protein in the ascitic fluid^5^, ^6^, ^7^. However, the serum albumin–ascites gradient (SAAG) criteria have completely replaced the traditional form of classification. According to these criteria, a value of ≥1.1 mg/dL is generally associated with increased portal pressure or portal hypertension (PH). However, the inability of the SAAG criteria to differentiate malignant ascites from other etiologies is a major problem.^7^ Ascitic fluid cytology is considered the standard test for malignancy, but its sensitivity is low.^8^ Both tuberculous peritonitis and malignant ascites have a similar chronic presentation, and the parameters tested (including tumor markers) give overlapping results.^9^, ^10^, ^11^, ^12^, ^13^, ^14^, ^15^ These conditions must be differentiated at an early stage because their treatment differs, meaning that early diagnosis could improve morbidity and associated mortality. Therefore, an alternative screening test is needed to differentiate ascites caused by malignancy from that caused by tubercular peritonitis.

Recently, the results of studies conducted to determine the accuracy of SAAG in discriminating between ascites related to PH and ascites not related to PH have called into question the sensitivity reported in early research.^15^, ^16^, ^17^ Furthermore, there have been no previous studies of Mexican patients that could demonstrate the true usefulness of SAAG, especially in those patients with serum albumin levels so low that they could be incorrectly classified within the group of pathologies that do not correspond to the level of SAAG. Thus, it would be desirable to establish a new cutoff level for SAAG that would allow the correct classification of all patients, especially those with very low serum albumin (<2.0 g/dL). This study aimed to identify a parameter that better classifies patients.

## Methods

### *Aims*

The purpose of this study was to evaluate the accuracy of the SAAG measure for the diagnosis of ascites secondary to PH in a Mexican population and use its sensitivity and specificity to establish cutoff points to predict PH.

### *Study design*

This was an analytical cross‐sectional study for the evaluation of the accuracy of a diagnostic test that was carried out between January 2018 and December 2018 in collaboration with the Biomedical Research Unit 02, Western National Medical Center, Mexican Institute of Social Security, and which included patients admitted with a diagnosis of ascites at the gastroenterology department of the Fray Antonio Alcalde Civil Hospital. Patients with a complete medical history who were admitted to the emergency department were included in the analysis.

Patients over 18 years old with a diagnosis of ascites of any etiology underwent complete studies (paracentesis, liver function tests, and serum albumin determination) for the determination of SAAG and albumin concentration in ascitic fluid (AFA). The analysis of serum albumin and ascitic fluid was performed using a Beckman Coulter AU5800 analyzer (Pasadena, CA, USA). Exclusion criteria were patients with a previous diagnosis of chylous ascites and severe coagulopathy or disseminated intravascular coagulation.

### *Sample size*

The sample size was calculated using a formula for discrete variables in cross‐sectional studies to identify the causes of PH in patients with ascites. For the desired errors α = 0.05 and β = 0.20, a minimum sample size of 85 patients was required.

### *Statistical analysis*

The descriptive phase of the analysis included the presentation of data as raw values, proportions, dispersion, and measures of central tendency. In the inference phase, the Student *t* test was used for continuous variables, and *χ* ^2^ or Fisher's exact test was used for qualitative variables when appropriate. Any value of *P* < 0.05 was considered significant.

To establish the SAAG cutoff values with the best sensitivity and specificity, a new cutoff point was identified using Receiver Operating Characteristic (ROC) curves. In addition, accuracy was determined by calculating sensitivity and specificity, and for safety, positive predictive value (PPV) and negative predictive value (NPV) were determined, and the accuracy of the test was calculated. The statistical analysis was performed using Excel 2007 (Microsoft, Redmond, WA, USA) and SPSS version 21 for Windows (IBM Corp., Armonk, NY, USA).

## Results

**Figure 1:**
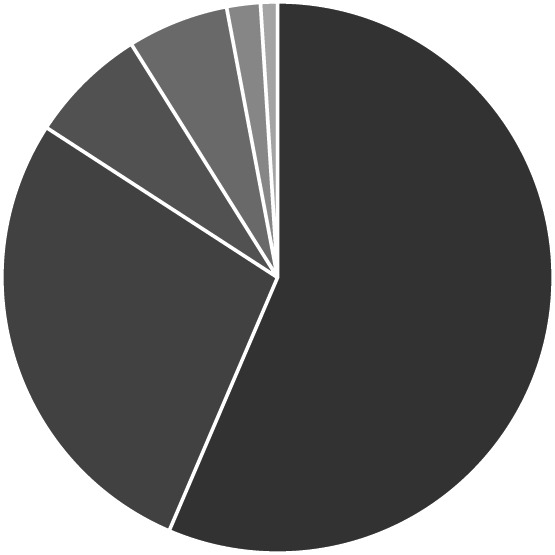
Frequency of each etiology of ascites diagnosis. (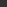), Alcohol; (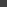), others (malignancy, peritoneal tuberculosis, spontaneous bacterial peritonitis) (28%); (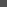), alcohol + hepatotropic virus (7%); (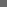), hepatotropic virus (6%); (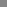), non‐alcoholic steatohepatitis (2%); (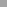), human immunodeficiency virus (1%).

**Figure 2:**
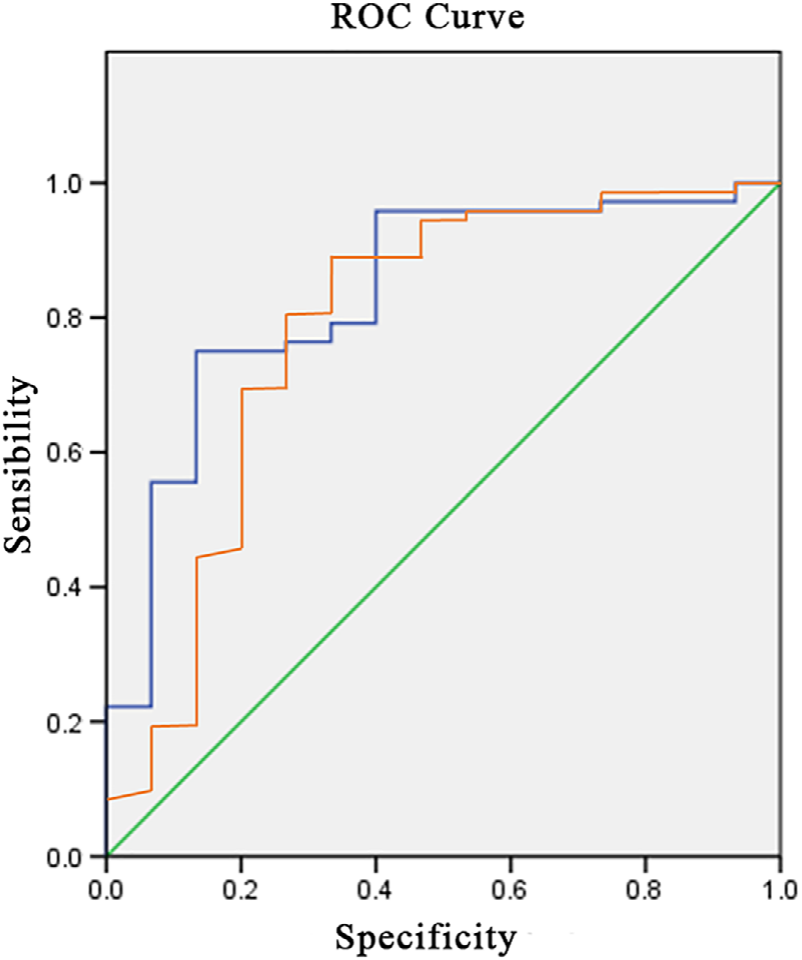
Comparison of receptor operating characteristics (ROC) curves for the diagnosis of portal hypertension ascites. (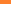), AFA, albumin concentration in ascitic fluid; (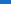), SAAG, serum–ascites albumin gradient.

During the study period, 95 patients met the criterion of clinical diagnosis of ascites of undetermined etiology and were admitted to the gastroenterology department of the Hospital Civil de Guadalajara Fray Antonio Alcalde. Of these patients, eight were excluded because they did not have a complete medical history or laboratory test results recorded. Finally, the study was conducted with 87 patients, 74 (84%) men and 13 (16%) women, with an average age of 54.1 ± 13.6 years. The frequency of each etiology of ascites diagnosed in the studied patients is shown in Figure 1. The general characteristics of the two groups of patients with PH‐related and non‐PH‐related ascites are shown in Table 1. By comparing the average result for each test, we can see that the average SAAG values and the AFA differ significantly between PH‐related and non‐PH‐related ascites. Of the 87 patients included in the analysis, 72 (83%) were admitted with a diagnosis of PH‐related ascites and 15 (17%) with non‐PH‐related ascites. A total of 50 patients had a SAAG ≥1.1, compared with 37 patients with a value <1.1. In the group with PH‐related ascites, 48 patients had a SAAG ≥1.1 g/dL and 24 <1.1 g/dL, compared with the patients with non‐PH‐related ascites, of whom 2 had a SAAG ≥1.1 g/dL, and 13 had a SAAG <1.1 g/dL. Regarding the AFA, we found that 23 patients had an AFA <1.1 g/dL, and 64 patients had an AFA ≥1.1 g/dL. In the group of patients with PH‐related ascites, 13 had an AFA ≥1.1 g/dL, while 59 had an AFA <1.1 g/dL. In comparison, the non‐PH‐related ascites group included 10 patients with an AFA ≥1.1 and 5 with an AFA value <1.1 g/dL. When comparing the usefulness of SAAG and AFA to diagnose PH‐related ascites, we found that SAAG correctly classified 48 (67%) of the 72 patients and incorrectly classified 24 (33%). In comparison, AFA correctly classified 59 (83%) and incorrectly classified 13 (17%). The diagnostic value of the two tests, including their sensitivity, specificity, PPV, and NPV using cutoffs of AFA <1.1 g/dL and SAAG ≥1.1 g/dL, are shown in Table 2. We observed that AFA had higher sensitivity but poor specificity, and SAAG had greater specificity but poor sensitivity. The evidence showed that there was a significant difference in the area under the ROC curves for SAAG and AFA (*P* < 0.001) (Fig. 2).

**Table 1:** General characteristics of patients diagnosed with portal hypertension (PH)‐related or non‐PH‐related ascites

Patient characteristics	PH	Non‐PH	*P‐*value	95% CI	OR
Total (*n*, %)	72 (82.7%)	15 (17.3%)			
Age (years)	52.6	58.7	0.08		
Gender (male/female)	63/9	11/4	0.15	0.666–9.728	2.54
Serum albumin (mean) (g/dL)	2.14	2.52			
SAAG (≥1.1, <1.1)	48/24	13/2	<0.001	2.71–62.31	13
AFA (mean) (g/dL)	0.79	2.46			
AFA (≥1.1, <1.1)	13/59	10/5	<0.001	2.65–31.05	9.07

AFA, albumin concentration in ascitic fluid; CI, confidence interval; OR, odds ratio; SAAG, serum–ascites albumin gradient.

**Table 2:** Results of the validation test for portal hypertension ascites

Test	Sensitivity (%)	Specificity (%)	PPV (%)	NPV (%)	Diagnostic accuracy (%)
SAAG	66	86	96	35	57
AFA	82	66	92	43	73

AFA, albumin concentration in ascitic fluid; NPV, negative predictive value; PPV, positive predictive value; SAAG, serum albumin–ascites gradient.

The SAAG in PH‐related ascites showed a cutoff value of 0.35, a sensitivity of 96%, and a specificity of 60% (95% confidence interval [CI]: 0.72–0.95). The AFA had a cutoff value of 2.27 with a sensitivity of 94% and specificity of 55% (95% CI: 0.63–0.93) in the same population.

## Discussion

Ascitic fluid accumulation in the peritoneal cavity can occur for a range of reasons that can be classified into two groups. The first is ascites related to PH, produced by increased portal circulation pressure or the development of splenic vasodilation. Hypoalbuminemia may also develop as a result of chronic liver disease that produces extravasation of fluid into the peritoneal cavity, decreasing the effective systemic circulating volume in response to increased water and sodium retention in the kidneys, making it a continuous process.^18^ The causes of PH‐related ascites are hepatic cirrhosis, portal venous thrombosis, and vascular hepatopathies such as Budd–Chiari syndrome.^19^

Non‐PH‐related ascites is mainly caused by local factors, such as increased protein permeability of capillaries, a discrepancy between lymph production and excretion, or a combination of these mechanisms, which can result in the production of exudative fluid in conditions such as peritoneal carcinomatosis, peritoneal tuberculosis, autoimmune diseases, and other inflammatory processes.^20^, ^21^ In addition, there are other causes of the production of exudative fluid, such as nephrotic syndrome or hypoalbuminemia produced by protein–calorie malnutrition.^19^, ^22^ Since the end of the 1980s, studies have focused on diagnosis, treatment planning, and determining the origin of the ascites based on analysis of the ascitic fluid and the value of SAAG.^23^ Thus, current management guidelines suggest that a SAAG value >1.1 is indicative of PH‐related ascites and a value <1.1 of non‐PH‐related ascites.

In 1995, Gupta *et al*.^24^ measured ascitic fluid total protein, albumin, cholesterol, the ascites/serum ratios of these parameters, and serum–ascites albumin and cholesterol gradients in 76 patients to assess their ability to differentiate cirrhotic, malignant, and tuberculous ascites. The specificity, sensitivity, and overall diagnostic accuracy of AFA were 100, 82, and 91%, respectively, while those of ascitic fluid (AF) total protein were 10076, and 88% respectively and of SAAG 91, 94, and 92%, respectively.

Prabhu *et al*. studied 60 patients^25^ and showed that SAAG had a sensitivity of 90% and specificity of 77.5% in differentiating cirrhotic from noncirrhotic ascites at a cutoff value of >1.1 g/dL, whereas it had a sensitivity of 85% and specificity of 70% in the differentiation of malignant from nonmalignant ascites at a cutoff value of 1.08 g/dL. The AFA levels had a sensitivity of 90% and specificity of 72.5% in differentiating malignant from nonmalignant ascites, at a cutoff value of >1.7 g/dL, whereas at a cutoff value of 0.8 g/dL, its sensitivity was 90% and specificity 90% for differentiating cirrhotic from noncirrhotic ascites. Rodríguez *et al*.^26^ evaluated the diagnostic accuracy of SAAG, protein concentration in the AF (AFTP), AFA, and the protein ascites/serum ratio (PASR) in 116 patients for the diagnosis of ascites due to PH. To determine whether ascites was caused by PH, they used cutoff values as follows: SAAG ≥1.1, AFTP <2.5, AFA <1.1, and PASR <0.5. The reported sensitivity and specificity of SAAG were 93 and 47%, respectively; for AFTP 80 and 89%, respectively; for AFA 85 and 87%, respectively; and for PASR 83 and 80%, respectively. The area under the ROC curve for SAAG was 0.70, for AFTP 0.84, PASR 0.81, and AFA 0.86.

In conclusion, we found that SAAG has low sensitivity but high specificity. This finding could be related to the remarkably low levels of albumin in our population, with the mean albumin levels of 2.1 g/dL in patients with PH. AFA ˂1.1 had a better sensitivity but quite a low specificity. The diagnostic accuracy of AFA was superior to that of SAAG for discriminating between PH‐related and non‐PH‐related ascites, so it could be used in clinical practice in isolation or together with SAAG to achieve a more accurate diagnostic approach. In addition, we established, for the first time, the cutoff points for both SAAG and AFA that showed the best sensitivity and specificity in the Mexican population.

Prospective and large‐scale evaluation is required in this and other settings to identify the usefulness of SAAG, identify its optimal cutoff points, and compare its diagnostic accuracy with that of other noninvasive markers.

1

## Data Availability

The datasets used and/or analyzed during the current study are available from the corresponding author on reasonable request.
